# Downregulation of A20 Expression Increases the Immune Response and Apoptosis and Reduces Virus Production in Cells Infected by the Human Respiratory Syncytial Virus

**DOI:** 10.3390/vaccines8010100

**Published:** 2020-02-24

**Authors:** María Martín-Vicente, Rubén González-Sanz, Isabel Cuesta, Sara Monzón, Salvador Resino, Isidoro Martínez

**Affiliations:** 1 Unidad de Infección Viral e Inmunidad, Centro Nacional de Microbiología, Instituto de Salud Carlos III, Majadahonda, 28220 Madrid, Spain; maria.martinv@externos.isciii.es (M.M.-V.); ruben.gsanz@gmail.com (R.G.-S.); sresino@isciii.es (S.R.); 2 Unidad de Bioinformática, Centro Nacional de Microbiología, Instituto de Salud Carlos III, Majadahonda, 28220 Madrid, Spain; isabel.cuesta@isciii.es (I.C.); smonzon@isciii.es (S.M.)

**Keywords:** respiratory syncytial virus, A20, ubiquitination, innate immune response, apoptosis

## Abstract

Human respiratory syncytial virus (HRSV) causes severe lower respiratory tract infections in infants, the elderly, and immunocompromised adults. Regulation of the immune response against HRSV is crucial to limiting virus replication and immunopathology. The A20/TNFAIP3 protein is a negative regulator of nuclear factor kappa B (NF-κB) and interferon regulatory factors 3/7 (IRF3/7), which are key transcription factors involved in the inflammatory/antiviral response of epithelial cells to virus infection. Here, we investigated the impact of A20 downregulation or knockout on HRSV growth and the induction of the immune response in those cells. Cellular infections in which the expression of A20 was silenced by siRNAs or eliminated by gene knockout showed increased inflammatory/antiviral response and reduced virus production. Similar results were obtained when the expression of A20-interacting proteins, such as TAX1BP1 and ABIN1, was silenced. Additionally, downregulation of A20, TAX1BP1, and ABIN1 increased cell apoptosis in HRSV-infected cells. These results show that the downregulation of A20 expression might contribute in the control of HRSV infections by potentiating the early innate immune response and increasing apoptosis in infected cells.

## 1. Introduction

Human respiratory syncytial virus (HRSV), which belongs to the *Pneumoviridae* family, is an important respiratory pathogen that affects the general population. However, severe infections, including bronchiolitis and pneumonia, occur mainly in infants, the elderly, and immunocompromised adults [1,2]. In children under five, HRSV produces more than 33 million lower respiratory tract infections globally each year, of which approximately 3 million require hospitalization, and 0.2% die [3,4]. Additionally, severe pediatric HRSV infections are associated with the development of long-term sequelae, such as wheezing and asthma [5]. HRSV also contributes to the exacerbation of asthma and chronic obstructive pulmonary disease in adults [6,7].

Despite its significant impact on global health, there is no vaccine or effective treatment against HRSV. For a long time it has been thought that an exuberant inflammatory response was behind HRSV pathogenesis [8,9,10]. However, in recent years it has been reported that a strong innate immune response protects against severe HRSV disease [11,12]. Understanding how the virus interacts with the infected cells might help to shed light on this issue and to uncover potential therapeutic targets to control virus replication and the associated immunopathology [13]. HRSV primarily infects epithelial cells lining the airways. These cells recognize virus infection through pattern recognition receptors (PRRs), which trigger intracellular signaling leading to the activation of the transcription factors nuclear factor kappa B (NF-κB), interferon regulatory factor 3 (IRF3) and IRF7, and the expression of inflammatory and antiviral genes [14]. These signaling pathways are tightly regulated to avoid excessive inflammation and tissue damage while ensuring virus clearance. Post-translational modifications, including ubiquitination/deubiquitination of several proteins involved in these pathways, are essential for fine-tuning regulation of the intracellular immune response [15,16].

Tumor necrosis factor α-induced protein (TNFAIP3), also termed A20, is a negative regulator of NF-κB and IRF3/7 transcription factors [17,18,19]. A20 has two catalytic activities: deubiquitinase, mediated by its N-terminal ovarian tumor domain (OTU), and E3 ubiquitin ligase, mediated by its C-terminal seven zinc-finger structure [20,21]. This unique characteristic provides A20 with the capacity to regulate not only NF-κB and IRF signaling, but also the Wnt pathway, cell death, and autophagy, among others [22]. Therefore, it is not surprising that polymorphisms in the *TNFAIP3* locus are involved in many inflammatory and autoimmune diseases, including rheumatoid arthritis, systemic lupus erythematosus, inflammatory bowel disease, juvenile idiopathic arthritis, coeliac disease, psoriasis, coronary artery disease in type 2 diabetes, systemic sclerosis, type I diabetes, and Sjogren’s syndrome [23]. To add to the complexity, in certain pathways, A20 acts at different junctures [24], and in some cases, its effects are independent of its catalytic activity [17,25]. Furthermore, the anti-inflammatory actions of A20 require its association with other proteins that determine its specificity and activity, such as Tax1-binding protein 1 (TAX1BP1), A20-binding inhibitor of NF-κB1 (ABIN1, also known as TNIP1), and the ubiquitin ligases ITCH and RING finger protein 11 (RNF11) [22].

A20 knockout (KO) mice die prematurely by cachexia and multiorgan inflammation [26], which highlights the vital immunoregulatory and anti-inflammatory functions of A20. In contrast, A20 deficiency only in myeloid cells protects mice against influenza A virus (IAV) infection, an effect mediated by increased cytokine and chemokine production [27]. The deletion of A20 in lung epithelial cells also leads to increased tolerance to IAV, although the exact mechanism of how this occurs is not clear [28]. These results suggest that treatments that regulate A20 expression may have a positive impact on IAV infections and possibly other respiratory viral infections.

In the present study, we investigated how A20 influences HRSV replication, and the subsequent cellular immune response, to identify potential targets that might help to control the virus infection.

## 2. Material and Methods

### 2.1. Cells and Virus

Human lung carcinoma cells (A549) and human carcinoma HeLa-derived cells (HEp-2) were grown in Dulbecco’s modified Eagle’s medium (DMEM, Hyclone, Logan, UT, USA) complemented with 10% fetal bovine serum (FBS, Biological Industries, Beit HaEmerk, Israel), 4 mM L-Glutamine (HyClone), 100 U/mL penicillin (Lonza, Verviers, Belgium) and 100 µg/mL streptomycin (HyClone) (DMEM10). Cells were cultured at 37 °C in a 5% CO_2_ atmosphere.

Viral stocks of the HRSV Long strain were obtained from clarified culture supernatants from HEp-2 infected cells by polyethylene glycol precipitation and centrifugation in a discontinuous sucrose gradient as previously described [29,30].

### 2.2. Viral Infections and Plaque Assays

A549 subconfluent monolayers were infected with HRSV at a multiplicity of infection (MOI) of 3 plaque-forming units (pfu) per cell in DMEM with 2% FBS (DMEM2) and incubated for 90 min at 37 °C. After this time, the inoculum was removed, and fresh DMEM2 was added. Samples (culture supernatants and cell pellets) were collected at different times post-infection.

HRSV titers were determined in HEp-2 cell monolayers inoculated with serial dilutions of the culture supernatants for 90 min at 37 °C and then overlaid with 0.7% agarose in DMEM2. Five days post-infection (dpi), the cell monolayers were fixed with 4% formaldehyde and permeabilized with methanol. Plaques were visualized by one-hour incubation with a mixture of monoclonal antibodies previously obtained in our laboratory against the HRSV glycoprotein G, glycoprotein F, and phosphoprotein [29], followed by one-hour incubation with an anti-mouse IgG horseradish peroxidase linked whole antibody (Abcam, Cambridge, UK), and 3-amino-9-ethylcarbazole (AEC, Alfa Aesar, Ward Hill, MA, USA). Virus plaques, which were visible to the naked eye, were counted and HRSV titers calculated.

### 2.3. Quantitative RT-PCR

Total RNA from mock-infected or infected cells was purified with the ReliaPrep RNA Cell Miniprep System (Promega, Madison, WI, USA) and was reverse-transcribed with the High-Capacity cDNA Reverse Transcription Kit (Applied Biosystems, Foster City, CA, USA) following the manufacturer’s instructions. Gene expression was analyzed in triplicate by quantitative RT-PCR (qRT-PCR) in a Step One instrument (Applied Biosystems, Foster City, CA, USA) following the manufacturer’s protocols. TaqMan MGB probes (FAM dye-labeled) for the following genes were used (Applied Biosystems): *actin-β (ACTB)* (Hs99999903_m1), *Tumor Necrosis Factor Alpha Induced Protein 3 (TNFAIP3)* (A20, Hs00234713_m1), *Tax1 Binding Protein 1 (TAX1BP1)* (Hs00195718_m1), *TNFAIP3 Interacting Protein 1 (TNIP1)* (ABIN1, Hs00374581_m1), *Itchy E3 Ubiquitin Protein Ligase (ITCH)* (Hs00230354_m1), *Ring Finger Protein11 (RNF11)* (Hs00702517_s1), *Interleukin 6 (IL-6)* (Hs00985639_m1), *Interferon-Stimulated Gene 15 (ISG15)* (Hs00192713_m1), *Tumor Necrosis Factor Alpha (TNF-α)* (Hs00174128_m1), *Interferon-β1 (IFN-β1)* (Hs01077958_s1), *Chemokine C-C motif ligand 5 (CCL5)* (Hs00982282_m1), *C-X-C Motif Chemokine Ligand 8 (CXCL8)* (IL-8, Hs00174103_m1), *Interleukin 1 Beta (IL-1β)* (Hs01555410_m1), and HRSV nucleoprotein (forward primer, 5′CATGATTCTCCTGATTGTGGGATGA3′; reverse primer, 5′TCACGGCTGTAAGACCAGATCTAT3′; probe, 5′CCCCTGCTGCCAATTT3′; Applied Biosystems).

Gene expression was normalized to *ACTB,* and relative quantifications were determined by the comparative CT (ΔΔCT) method.

### 2.4. Western Blots

Protein expression was analyzed by Western blotting as previously described [31] using the following primary antibodies: anti-β-actin (Ab8224, Abcam), anti-A20 (Ab92324, Abcam) and anti-HRSV nucleoprotein (79N) [32].

### 2.5. siRNA Silencing

A549 cells were seeded 24 h before transfection at a density of 4.5 × 10^4^ cells per well in 24-well plates. Twenty-four hours later, cells were transfected with 6 pmol of control small interfering RNAs (siRNAs) (negative control #2) or specific siRNAs against *TNFAIP3* (A20, ID # s14259), *TAX1BP1* (ID # s16984), *ABIN1* (*TNIP1*, ID # s20174), *ITCH* (ID # s38163) and *RNF11* (ID # s25671) (all siRNAs were purchased from Ambion-Thermo Fisher, Rockford, IL, USA) and 1 µl of Lipofectamine RNAiMAx reagent (Invitrogen) per well. Twenty-four hours after transfection, cells were infected with HRSV at a MOI of 3. Cell supernatants for viral titration and cell pellets for RNA and protein extraction were harvested at different hours post-infection (hpi), as indicated in the figure legends.

### 2.6. A20 Knockout A549 Cells

Two clones of A20 knockout (A20^-/-^) A549 cells were produced using the Transcription Activator-Like Effector Nucleases (TALENs) technology [33] and the plasmids Human-H27583_TNFAIP3_TALEN-L and Human-H27583_TNFAIP3_TALEN-R (Talen Library Resource, Seoul National University), as previously described [31]. In brief, A549 cells (parental cells) were transfected with those plasmids encoding sequence-specific DNA-cleaving nucleases against A20. Three days after transfection, the cells were trypsinized and cloned by limiting dilution in 96-well plates at a density of 1 cell per well. Single-cell clones were selected, cloned a second time by limiting dilution, and expanded to generate stocks. Screening for A20^-/-^ clones was done by PCR amplification and DNA sequencing using the following primers: forward (5′- CCTTTGCAACATCCTCAGAAG-3′) and reverse (5′- ACTAACCAAGCAAGTCACAGAAC-3′). A20^-/-^ clones were checked by Western blotting using an A20 specific antibody (Ab167154, Abcam, Cambridge, UK). Two A20^-/-^ clones (KO-1 and KO-2) were selected. In addition, one wild-type (A20^+/+^) clone (WT-1) that underwent the same process of transfection and cloning, but in which the A20 gene was not disrupted, was selected as control (Supplementary Materials, Figure S1).

### 2.7. Overexpression Assays

Plasmids encoding human TNFAIP3 (pFlag-A20) and TAX1BP1 (pTAX1BP1) were kindly provided by Edward W. Harhaj (Penn State Cancer Institute, Hershey, PA, USA) [34,35,36,37]. Plasmid encoding human TNFAIP3 interacting protein 1 (TNIP1) (Myc-DDK-tagged, accession No. NM_006058) was purchased from Origene (Origene, Rockville, MD, USA). A control plasmid was generated by restriction enzyme digestion of the TNFAIP3 plasmid to release the insert and subsequent ligation of the vector.

For overexpression assays, 1.6 × 10^5^ A549 cells were plated in each well of a 12-well plate and incubated for 24 h before transfection. Cells were then transfected with 1 µg of the purified plasmid (EndoFree Plasmid Maxi Kit, Qiagen, Hilden, Germany) and 3 µl of Lipofectamine 3000 (Invitrogen, Carlsbad, CA, USA) per well. Twenty-four hours after transfection, the cells were infected with HRSV at a MOI of 3. Cell supernatants for viral titration and cell pellets for RNA and protein extraction were collected at different hpi.

### 2.8. Apoptosis Assay

Cell apoptosis was measured by the Single Channel Dead Cell Apoptosis Kit with Annexin V-Alexa-fluor488 and Sytox green dyes from Thermo Fisher (cat. # V13240) following the manufacturer’s instructions. Fluorescence was detected in a BD FACSCanto^TM^ Flow Cytometer (BD Biosciences, San José, CA, USA) and analyzed by the FlowJo^TM^ software (BD Biosciences).

### 2.9. Stranded mRNA-seq Library Preparation and Sequencing

Parental A549 cells and KO-1 and KO-2 cell lines were infected with HRSV at a MOI of 3. Twenty-four hours later, total RNA was extracted from infected cells with the ReliaPrep RNA Cell Miniprep System (Promega, Madison, WI, USA) following the manufacturer’s instructions. RNA was quantified with a BioPhotometer Plus (Eppendorf, Hamburg, Germany) and RNA quality was assessed with the 2100 Bioanalyzer RNA NANO assay (Agilent Technologies, Santa Clara, CA, USA). All samples had an RNA integrity number (RIN) higher than nine (Supplementary Materials, Figure S2). Libraries were prepared using the TruSeq Stranded mRNA Sample Prep Kit v2 (ref. RS-122-2101/2, Illumina) according to the manufacturer’s protocol. Briefly, 500 ng of total RNA was used for poly(A)-mRNA selection using Oligo (dT) magnetic beads and were subsequently fragmented to approximately 300bp. cDNA was synthesized using reverse transcriptase (SuperScript II, ref. 18064-014, Invitrogen) and random primers. The second strand of the cDNA incorporated dUTP in place of dTTP. Double-stranded DNA was further used for library preparation. dsDNA was subjected to A-tailing and ligation of the barcoded Truseq adapters. All purification steps were performed using AMPure XP Beads (ref. A63881, Beckman Coulter). Library amplification was performed by PCR using the primer cocktail supplied in the kit.

Final libraries were analyzed using Agilent DNA 1000 chip to estimate the quantity and check size distribution and were then quantified by qPCR using the KAPA Library Quantification Kit (ref. KK4835, KapaBiosystems) before amplification with Illumina’s cBot. Libraries were sequenced using 50 base read lengths in single-end mode (1 × 50) on Illumina’s HiSeq 2500.

### 2.10. Data Analysis

The obtained RNA-Seq data were analyzed by the Bioinformatics Facility of the Instituto de Salud Carlos III (ISCIII). First, quality control analysis involving FastQC (v0.11.3) [38] was carried out, and any adapter sequences, as well as low-quality 3′ ends were removed using Trimmomatic v0.36 [39]. A sliding window quality filtering approach was performed, scanning from the 5′ end of the read, and removing the 3′ end of the read when the average quality dropped below a Q score of 15. Reads lower than 50 nucleotides in length were removed.

The high-quality reads were then mapped against Hg38 human genome using Tophat (v2.0.14) [40,41], and mapping quality control was performed using RseQC (v2.6.4) [42,43].

Transcriptome prediction and gene/isoform quantification were calculated using Cufflinks (v2.2.1) based on Hg38 Ref-Seq reference genes [44,45]. Finally, differential expression analysis was carried out using Cuffdiff, which uses Benjamini–Hochberg correction to compute the False Discovery Rates (FDR) (q-value). Differentially expressed genes (DEGs) were considered when FDR values were ≤0.05. The CummeRbund package (v2.14.0) was used for quality control and visualization of the results.

### 2.11. Functional Annotation of Candidate Genes

KO cells and parental A549 cells were infected at a MOI of 3. At 24 hpi, RNA was extracted and processed for sequencing. Data from three independent biological experiments were used to generate two lists of differentially expressed genes (FC ≥ 1.5, q ≤ 0.05) by comparing KO-1 or KO-2 cells vs. the A549 parental cells. These two lists were compared and the genes that were upregulated in both KO lines were selected (Supplementary Materials, File S1). Functional annotation of selected genes was performed using DAVID (Database for Annotation, Visualization and Integrated Discovery) Bioinformatics Resources 6.8 [46,47], applying an EASE score threshold of 0.1 and a count threshold of 2.

### 2.12. Statistical Analysis

Pairwise comparisons between control and test conditions were done by using a Student’s *t*-test to determine statistically significant differences. Statistics were calculated with the Graphpad Prism 8 software. From the p values obtained, q-values (FDR) were calculated using the Benjamini–Hochberg method and indicated in the figures as * (q-values < 0.05) and ** (q-values < 0.01). Means and standard deviations from at least three independent experiments are represented in the figures.

## 3. Results

### 3.1. HRSV Infection Induces High Levels of A20 Expression

**Figure 1:**
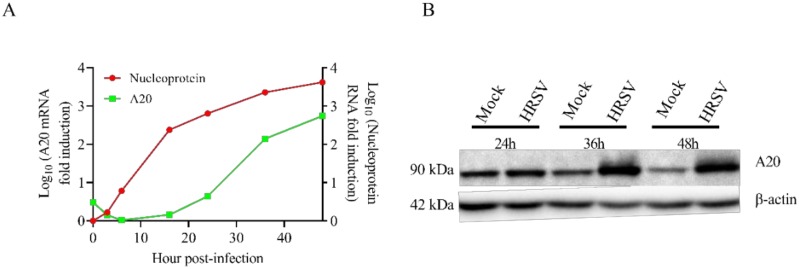
Human respiratory syncytial virus (HRSV) infection of A549 cells enhances A20 expression. A549 cells were infected with HRSV at a multiplicity of infection (MOI) of 3 and harvested at the indicated times post-infection. (**A**) HRSV nucleoprotein and A20 RNAs were quantified by qRT-PCR. The data represent the fold increase of A20 RNA in infected cells compared with mock-infected cells and the fold increase of HRSV nucleoprotein RNA with respect to 0 h post-infection (hpi). RT-qPCRs at each time point were done in triplicate wells. (**B**) Protein accumulation was analyzed by Western blotting using an anti-A20 specific antibody. Normalization was carried out using an anti-β-actin antibody.

To explore if A20 expression was upregulated during HRSV infection, A549 cells were infected, and the levels of A20 and HRSV nucleoprotein (N) RNAs were measured by qRT-PCR at different times post-infection. A large amount of A20 mRNA was produced in infected cells at later times post-infection (36–48 h post-infection) (Figure 1A). The expression of A20 was delayed with respect to HRSV N. While levels of HRSV N started to increase after 3-6 hpi, the accumulation of A20 mRNA was only detected following 16-24 hpi, reaching levels of about 500 times over uninfected cells by 48 hpi (Figure 1A).

To confirm that the upregulation of A20 mRNA correlated with an increase at the protein level, the amounts of A20 protein were analyzed by Western blotting at different times post-infection. The results confirmed that A20 protein was accumulated in HRSV infections after 24 hpi (Figure 1B).

### 3.2. Downregulation of A20, TAX1BP1, or ABIN1 Enhances Intracellular Immune Response and Decreases HRSV Virus Production

Since A20 is a negative regulator of NF-κB and IRF3/7 transcription factors, which are key factors in the inflammatory and antiviral responses, we analyzed the impact of A20 downregulation on the expression of different cytokines and chemokines in cells infected with HRSV. Also, downregulation of some A20-interacting proteins that modulate A20 activity and specificity was tested. Gene silencing was confirmed by RT-PCR and the results are shown in Supplementary Materials, Figure S3.

**Figure 2:**
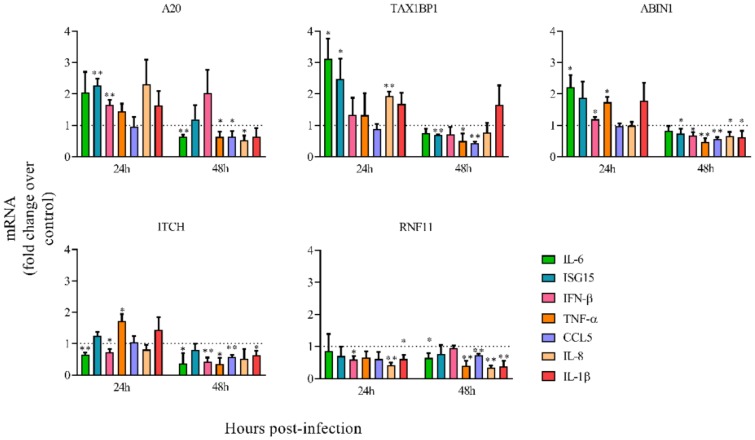
Relative mRNA levels of inflammatory/antiviral genes in cells transfected with different siRNAs. Cells were transfected with the indicated siRNAs (A20, TAX1BP1, ABIN1, ITCH, RNF11) and infected 24 h later at a MOI of 3. Levels of mRNAs were quantified by qRT-PCR at 24 and 48 hpi and represented as fold-over mRNAs expressed in cells transfected with a control siRNA and infected 24 h later at a MOI of 3. Data are represented as the mean and standard deviation from three independent experiments. Pairwise comparisons between cells transfected with a specific siRNA and cells transfected with the control siRNA, were done for each gene and time post-infection by using the *t*-test. From the p values obtained, False Discovery Rates (FDRs) were calculated: *, q-value < 0.05 and **, q-value < 0.01. Fold change = 1 means that there is no expression change with respect to the control (cells transfected with a siRNA control) and is represented by a dotted line.

siRNA-mediated silencing of A20 led to an increase in the expression at 24 hpi of all immune genes tested, except CCL5 (Figure 2). Similar results were obtained when TAX1BP1 or ABIN1 were downregulated (Figure 2), in accordance with previous reports indicating that A20, TAX1BP1, and ABIN1 collaborate to inhibit antiviral signaling [48]. However, only ISG15, TNF-α and IL-1β were overexpressed in ITCH-silenced cells, and none of the immune genes tested was upregulated in RNF11-silenced cells (Figure 2). In contrast, the expression of the majority of the immune genes tested was slightly downregulated in all cases at 48 hpi (Figure 2).

**Figure 3:**
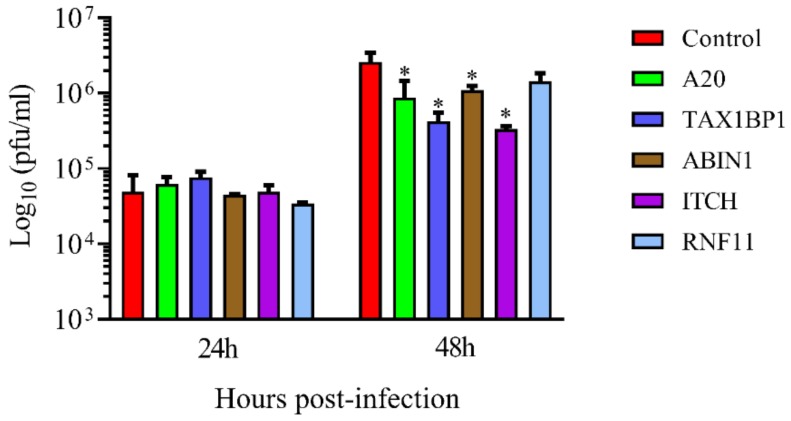
Human respiratory syncytial virus titers in the supernatant of infected cells transfected with different siRNAs. Cells were transfected with the siRNAs (control, A20, TAX1BP1, ABIN1, ITCH, RNF11) and infected 24 h later at a MOI of 3. Supernatants were collected at 24 and 48 hpi, and the virus was titrated by plaque assay. Data are represented as the mean and standard deviation from three independent experiments. Pairwise comparisons between cells transfected with a specific siRNA (A20, TAX1BP1, ABIN1, ITCH or RNF11) and cells transfected with a control siRNA, were done for each time post-infection by the *t*-test. From the p values obtained, FDRs were calculated. *, q-value < 0.05.

To test if the downregulation of these mRNAs had any effect on HRSV growth, virus titers in the supernatant of silenced cells were measured. Again, A20, TAX1BP1, and ABIN1 silencing reduced virus titers significantly (Figure 3 and Supplementary Materials, Table S1). RNF11 downregulation did not affect HRSV titers, but ITCH downregulation reduced virus titers by approximately ten times (Figure 3 and Supplementary Materials, Table S1).

### 3.3. HRSV Production is Impaired in A20 Knockout Cells While the Antiviral/Inflammatory Response Is Augmented in These Cells

To confirm that A20 is involved in the regulation of the early innate immune response against HRSV, two A20 knockout lines were generated from A549 cells (KO-1 and KO-2). As a control, one wild-type cell line was generated via the same process of cloning (WT-1). The lack of expression of A20 protein in KO cells was checked by Western blotting (Supplementary Materials, Figure S1).

**Figure 4:**
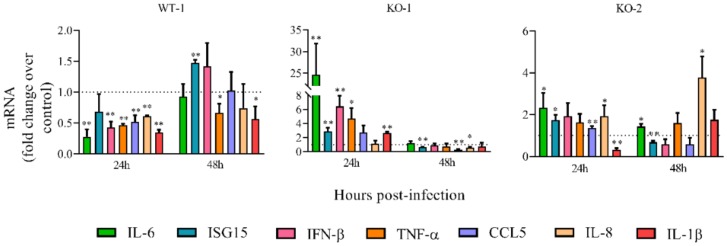
Relative mRNA levels of inflammatory/antiviral genes in A20 knockout (KO) cells. Cells were infected at a MOI of 3. Levels of mRNAs were quantified by qRT-PCR at 24 and 48 hpi and represented as fold-over mRNAs expressed in infected A549 parental cells. Data are represented as the mean and standard deviation from three independent experiments. Pairwise comparisons between each cell type and parental A549 cells (control) were done for each gene and time post-infection by using the *t*-test. From the p values obtained, FDRs were calculated: *, q-value < 0.05 and **, q-value < 0.01. KO-1 and KO-2, A549 cell lines that do not express A20. WT-1, wild-type A549 cell line obtained following the same process as with KO-1 and KO-2. Fold change = 1 means that there is no expression change with respect to the control (A549 cells) and is represented by a dotted line.

Cell lines were infected with HRSV, and the expression of inflammatory/antiviral genes was compared to infected uncloned A549 wild-type cells. Similar to what happened in the A20 silenced cells, most of the immune genes tested were significantly upregulated in both KO lines at 24 hpi (Figure 4). Only IL-1β was downregulated at this time in the KO-2 line (Figure 4). At 48 hpi, however, the majority of genes not only were not upregulated, but were even downregulated (Figure 4), also resembling the results observed in A20 silenced cells (Figure 2). Only IL-6 and IL-8 were significantly upregulated at 48 hpi in KO-2 (Figure 4). Unlike the KO lines, the wild-type line (WT-1) showed decreased expression of the immune genes at 24 hpi (Figure 4) and variable expression at 48 hpi (Figure 4).

To further confirm the role of A20 in the regulation of the early innate immune response following HRSV infection, we restored the expression of A20 in the KO cell lines by transfection with plasmids encoding this protein. As expected, the restoration of A20 expression in KO-2 cells decreased the expression of most of the antiviral/inflammatory genes at 24 hpi when compared to KO-2 cells transfected with a control plasmid (empty vector) (Supplementary Materials, Figure S4C). However, this effect was not observed in the KO-1 cells (Supplementary Materials, Figure S4B), probably because, for unknown reasons, the expression of A20 in KO-1 cells was much lower than in KO-2 cells (Supplementary Materials, Figure S4A).

**Figure 5:**
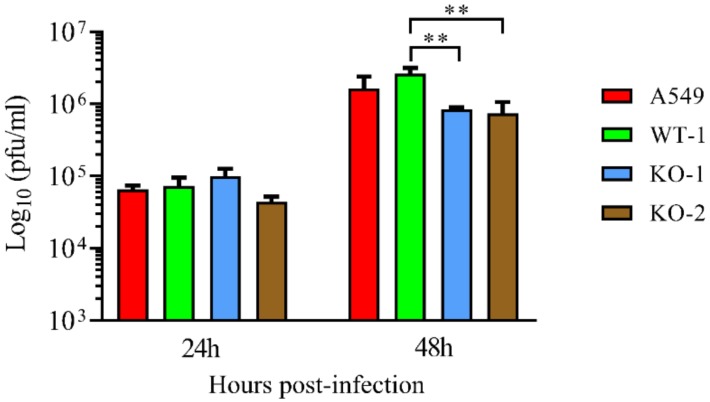
Human respiratory syncytial virus titers in the supernatant of A20 KO cells. Cells were infected at a MOI of 3. Supernatants were collected at 24 and 48 hpi and the virus was titrated by plaque assay. Data are represented as the mean and standard deviation from three independent experiments. Statistically significant differences between indicated cell lines were determined by the *t*-test. From the p values obtained, FDRs were calculated: ** q-value < 0.01. KO-1 and KO-2, A549 cell lines that do not express A20. WT-1, wild-type A549 cell line obtained following the same process as with KO-1 and KO-2.

Since A20 silencing led to a decrease in HRSV titers, the production of infectious HRSV was analyzed in the KO lines. Like with A20 silenced cells, virus titers were reduced in both KO lines (from two to three times) when compared to A549 cells and the WT-1 line at 48 hpi (Figure 5 and Supplementary Materials, Table S2).

### 3.4. Immune Response and Infection-Related Signaling Pathways Are Upregulated in A20 Knockout Cells Following HRSV Infection

To further confirm that A20 is involved in the regulation of the intracellular immune response against HRSV, uncloned wild-type A549 cells and the A20 KO lines were infected with the virus, and the expression of cellular genes was analyzed by RNAseq. Data from three independent biological experiments (infections) were analyzed to obtain a list of genes that were upregulated (FC ≥ 1.5; q ≤ 0.05) in both KO-1 and KO-2 cell lines (Supplementary Materials, File S1). This gene list was analyzed using the DAVID Functional Annotation Tool (see Material and Methods). Gene Ontology Biological Processes (GO-BP) related to immune response were over-represented in both KO lines when compared to A549 cells (Table 1). Similarly, pathways related to immune response and anti-microbial response were also over-represented in the KO lines (Table 2).

### 3.5. Downregulation of A20 and A20-Interacting Proteins Increases Cell Apoptosis in HRSV-Infected Cells

In addition to inflammation, A20, TAX1BP1, ABIN1, and ITCH have been shown to inhibit apoptosis [22,49,50,51,52]. Since HRSV induces cell apoptosis at later times post-infection [53,54], we investigated if the downregulation of these proteins had any effect on cell apoptosis after 48 hpi.

**Figure 6:**
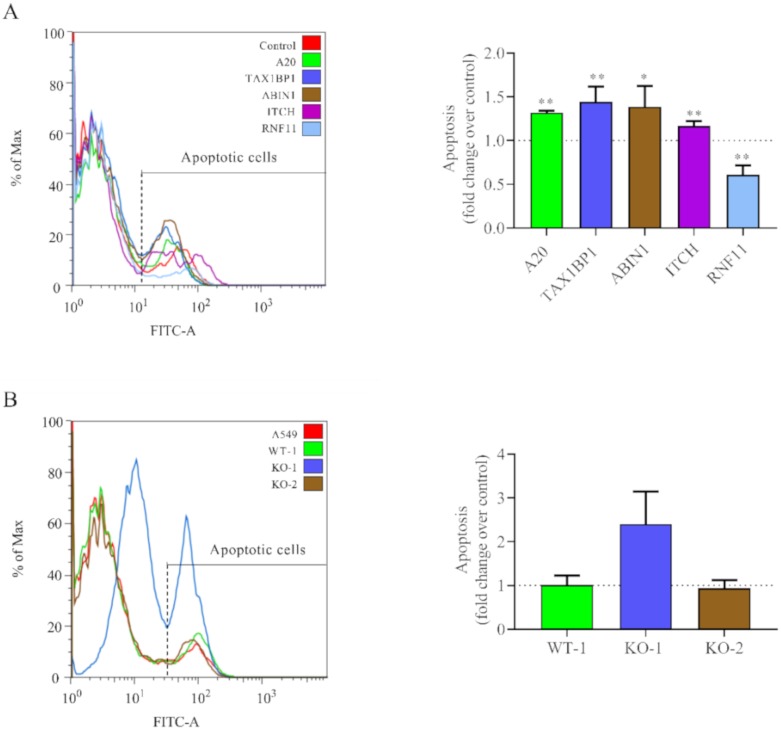
Apoptosis levels in siRNA-treated or A20 KO cells. (**A**) Cells were transfected with the indicated siRNAs (control, A20, TAX1BP1, ABIN1, ITCH, and RNF11) and infected 24 h later at a MOI of 3. (**B**) Wild-type and A20 KO cells were infected at a MOI of 3. In all cases, cells were harvested at 48 hpi and apoptosis was quantified via flow cytometry by AnnexiV-AlexaFluor488^TM^ and Sytox^TM^ staining. The left panels show results from one representative experiment, and the right panels represent the mean (fold-over control) and standard deviation from three independent experiments. Controls are siRNA control in (**A**) and parental A549 cells in (**B**). Pairwise comparisons between cells treated with the siRNA control and a specific siRNA (**A**) or A549 cells with the cell lines (WT-1, KO-1 or KO-2) (**B**) were done by the *t*-test. From the p values obtained, FDRs were calculated: *, q-values < 0.05 and **, q-values < 0.01.

Gene silencing showed that downregulation of A20, TAX1BP1, ABIN1, and ITCH but not RNF11, increased apoptosis in cells infected with HRSV at later times post-infection (Figure 6A). Similarly, enhanced apoptosis was observed in the A20 KO-1 cell line following virus infection (Figure 6B). However, no differences were detected between the KO-2 cell line and wild-type cells (Figure 6B).

## 4. Discussion

In this study, we show that A20 is involved in the regulation of the innate immune response and apoptosis in HRSV-infected epithelial cells. Downregulation of A20 by specific siRNAs or the lack of A20 expression in KO cells led to increased inflammatory/antiviral response and apoptosis after HRSV infection. A decrease in virus titers was also observed. At least two A20-interacting proteins, TAX1BP1 and ABIN1, seem to cooperate in that regulation since similar effects were observed in cells in which the expression of those proteins was downregulated by siRNA treatment.

The induction of the inflammatory/antiviral response in HRSV-infected epithelial cells mostly depends on the activation of the retinoic acid-inducible gene I (RIG-I) receptor by viral RNA and subsequent activation of the NF-κB and IRF3 transcription factors [55]. For example, the activation of both NF-κB and IRF3 by HRSV infection, as well as the expression of several cytokines and antiviral genes, appear to be inhibited when RIG-I is downregulated by siRNAs [55] (our unpublished results). A20 regulates the activity of NF-κB and IRF3/7 in response to multiple stimuli, including RIG-I activation by viral RNAs [22]. A20 interrupts RIG-I signaling at several junctures by ubiquitination/deubiquitination of some proteins that participate in the signaling pathway [24,56]. Non-catalytic mechanisms, such as A20 competing with other ubiquitin-binding proteins like NEMO to limit IKK phosphorylation and downstream NF-κB activation have also been observed [57,58,59,60]. Some of these roles of A20 require that the interactions with partner proteins are functional. For instance, A20 cooperates with TAX1BP1 and ABIN1 to disrupt the TRAF3-TBK1-IKKε complex, thereby inhibiting IRF3 activation [48,61]. In agreement with this, we observed the upregulation of the innate immune genes in HRSV-infected cells silenced for A20, TAX1BP1 or ABIN1, showing the importance of those proteins in regulating the inflammatory/antiviral immunity against this virus [24]. In contrast, RNF11 silencing did not result in increased expression of immune genes, apoptosis, or HRSV growth, indicating that this protein does not collaborate with A20 in the regulation of these processes, despite the fact that RNF11 seems to form a complex with A20, TAX1BP1, and ITCH to inhibit TNF and LPS-induced NF-κB signaling [62]. Results from ITCH silencing are less easy to interpret since its impact on the innate immune response against HRSV appeared to be less than that of A20, TAX1BP1 or ABIN-1 silencing, while the reduction in viral titers was similar or even higher. It has been shown that TAX1BP1 recruits ITCH to the mitochondrial adaptor MAVS to promote its ubiquitination and degradation and thus restricts virus-induced apoptosis [50]. The increased apoptosis seen in ITCH-silenced cells may explain, at least in part, the reduced HRSV replication observed in our study, as discussed below.

Overall, the results obtained on A20 silencing were confirmed in the studies on HRSV infections of A20 KO cells. However, the two A20 KO lines differed significantly from each other and from A20-silenced wild-type cells in regards to the innate immune response after HRSV infection. It should be taken into account that the KO lines were established from a single cell after multiple rounds of replication. In this process, different epigenetic changes may have occurred in the two KO lines to compensate, to some extent, for the A20 deficiency.

Overexpression experiments were conducted to confirm the results obtained in KO cells and via siRNA silencing. Although we transfected cells with single, or triple combinations of plasmids expressing A20, TAX1BP1, or ABIN1, we were unable to see differences from cells transfected with control plasmids in regards to the expression of immune genes or HRSV replication (Supplementary Materials, Figure S5). Overexpression experiments are more challenging to perform than siRNA silencing, especially when several genes have to be expressed in a single cell to reconstruct a protein complex. Furthermore, since A20 can interact with multiple partners to carry out different functions [34,36,62,63], other unidentified proteins, in addition to A20, TAX1BP1, and ABIN1, may form part of the complex required for the regulation of the intracellular innate immune response against HRSV.

In the present study, downregulation of A20, TAX1BP1, and ABIN1 expression led to an increased immune response in HRSV-infected cells at 24 hpi, but not at 48 hpi. There are at least two possible explanations for this result. First, although A20 negatively regulates the canonical NF-κB pathway, it has been reported that it also activates the non-canonical pathway by a non-catalytic mechanism [64]. In that study, the authors propose that A20 may be involved in the molecular switch from canonical to non-canonical activation of NF-κB pathways. The non-canonical route is activated in HRSV-infected cells [65], and we have shown that genes involved in this pathway, including *NFKB2*, *RELB*, and *NIK*, are upregulated at later times post-infection [29]. It is possible that at 48 hpi, the activation of the canonical pathway due to a lack of A20-related activity in silenced cells may be compensated by the inhibition of the non-canonical route. However, it has not been elucidated if the activation of the non-canonical pathway by A20 requires the cooperation of TAX1BP1 and ABIN1. To add to the complexity, Liu et al. [66] showed that in addition to the canonical and non-canonical pathways, a “cross-talk pathway” operates in HRSV-infected cells downstream of RIG-I, leading to the activation of the NIK-IKKα complex and RelA release. Second, downregulation of A20, TAX1BP1 and ABIN1 decreased virus production at later times post-infection, and this may have contributed to reducing the intracellular immune response at this time. Inhibition of HRSV replication has been shown to decrease the inflammatory/antiviral response in infected cells [67].

We have shown that A20 is highly expressed at later times after HRSV infection of epithelial cells. This is in line with the idea that one of the central functions of A20 is to contribute to the termination of the immune response to avoid immune-mediated tissue damage. However, the upregulation of A20 may also be beneficial for the virus. Thus, an interesting observation of our study is that viral replication was reduced at 48 hpi in cells treated with siRNAs against A20 or A20-interacting proteins, as well as in A20 KO cells. This may be a result of the increased antiviral response observed in those cells. In line with this, it has been described that A20 promotes influenza virus replication by suppressing the antiviral response of infected cells [68]. Similarly, it has been shown that A20 promotes hepatitis C virus and human cytomegalovirus replication [69,70,71]. Additionally, the increased apoptosis observed in silenced and KO cells may have also contributed to reducing virus production. Apoptosis is a mechanism of programmed cell death that can function to restrict microbial growth [72]. In agreement with this, it has been reported that inhibition of apoptosis promotes HRSV replication [73,74]. Therefore, the strong upregulation of A20 in HRSV-infected cells may facilitate virus replication and persistence by decreasing antiviral response and apoptosis [75].

## 5. Conclusions

In conclusion, our data indicate that A20, probably with the collaboration of other protein partners, modulates the intracellular innate immune response and apoptosis in HRSV-infected epithelial cells, which affects virus production. Therefore, A20 and/or A20-interacting proteins may be potential targets to regulate two crucial aspects of HRSV infections, namely, virus growth and the early inflammatory/antiviral response.

## Figures and Tables

**Table 1:** List of the top ten Gene Ontology Biological Processes over-represented in the genes that are up-regulated in both the infected KO-1 and KO-2 cells.

Term	Gene Symbol	Benjamini (*p*-Value)
GO:0006955 Immune response	CCL26, CXCL2, CXCL5, CXCL8, CD70, CD74, SAMHD1, TNFRSF1B, TNFRSF21, B2M, CTSS, CIITA, C1R, C3, GBP2, IFITM2, IL1RAP, IL7R, HLA-C, PDCD1LG2, TLR4, TRIM22, TNFSF10, TNFSF13B, TNFSF14, TNFSF9	0.017
GO:0001666 Response to hypoxia	CD24, CD38, CASP1, DPP4, EGLN1, EDNRA, HIF1A, LOXL2, MMP2, MUC1, PLAT, PLOD2, SLC2A8, SOD2, TGFBR2	0.031
GO:0051607 Defense response to virus	MX2, SAMHD1, APOBEC3G, GBP1, GBP3, IFI16, IFI44L, IFIT1, IFITM1, IFITM2, IL33, SERINC5, TLR3, TRIM22	0.055
GO:0006954 Inflammatory response	CCL17, CCL26, CXCL2, CXCL5, CXCL8, SMAD1, TNFRSF1B, TNFRSF21, AOX1, CHST2, CASP4, CIITA, C3, ITGB2, IFI16, IL1RAP, NR1H4, PTX3, RPS6KA5, THEMIS2, TLR3, TLR4	0.060
GO:0060333 Interferon-gamma-mediated signaling pathway	IFI30, B2M, CIITA, GBP1, GBP2, IFNGR1, HLA-C, MT2A, TRIM22	0.069
GO:0007155 Cell adhesion	ADAM12, CD24, EDIL3, EPHA4, KIAA1462, KITLG, CDH2, CDH6, CEACAM1, COL4A6, COL5A1, EFNB2, FAP, FN1, LGALS3BP, ISLR, IGFBP7, ITGB2, LSAMP, LOXL2, RGMB, SCN1B, TNC, THEMIS2	0.084
GO:0060337 Type I interferon signaling pathway	MX2, SAMHD1, XAF1, GBP2, IFIT1, IFITM1, IFITM2, HLA-C	0.15
GO:0002237 Response to molecule of bacterial origin	CXCL2, CXCL8, CD24, MALT1	0.20
GO:0045766 Positive regulation of angiogenesis	CXCL8, GATA2, GATA6, WNT5A, CCBE1, C3, HIF1A, ITGB2, TGFBR2, TWIST1	0.22
GO: 0040037 Negative regulation of fibroblast growth, factor receptor signaling pathway	WNT4, WNT5A, SPRY1, SULF2	0.22

**Table 2:** Kyoto Encyclopedia of Genes and Genomes (KEGG) pathways over-represented in the genes that are up-regulated in both the infected KO-1 and KO-2 cells.

Term	Gene Symbol	Benjamini (*p*-Value)
hsa04060 Cytokine-cytokine receptor interaction	CCL17, CCL26, CXCL2, CXCL5, CXCL8, CD70, TNFRSF19, TNFRSF1B, TNFRSF21, IFNGR1, IL1RAP, IL7R, LIFR, TGFBR2, TNFSF10, TNFSF13B, TNFSF14, TNFSF9	0.015
hsa05133 Pertussis	CXCL5, CXCL8, CASP1, C1R, C1S, C3, C4BPA, ITGB2, TLR4	0.041
hsa05134 Legionellosis	CXCL2, CXCL8, CASP1, C3, EEF1A2, ITGB2, TLR4	0.11
hsa00760 Nicotinate and nicotinamide metabolism	NT5C3A, CD38, AOX1, NNMT, NNT	0.21
hsa04610 Complement and coagulation cascades	C1R, C1S, C3, C4BPA, CFB, CFI, PLAT	0.22
hsa05150 Staphylococcus aureus infection	C1R, C1S, C3, CFB, CFI, ITGB2	0.27
hsa05152 Tuberculosis	CD74, MALT1, CTSS, CIITA, C3, EEA1, ITGB2, IFNGR1, TLR4, VDR	0.55
hsa04145 Phagosome	CTSS, C1R, C3, EEA1, ITGB2, HLA-C, TLR4, TUBA1A, TUBB3	0.51
hsa04612 Antigen processing and presentation	CD74, IFI30, B2M, CTSS, CIITA, HLA-C	0.55
hsa05146 Amoebiasis	CXCL8, COL4A5, COL4A6, COL5A1, FN1, ITGB2, TLR4	0.58

## Supplementary Materials

The following are available online at https://www.mdpi.com/2076-393X/8/1/100/s1, Figure S1: Western blotting of A20-knockout cell lines, Figure S2: RNA quality parameters, Figure S3: Gene silencing of A20, TAX1BP1, ABIN1, ITCH, and RNF11 genes, Figure S4: Overexpression of A20 in A20-knockout cell lines, Figure S5: HRSV titers and immune gene expression in cells transfected with individual plasmids or a mixture of plasmids encoding A20, TAX1BP1 and ABIN1, Table S1: Raw values of virus titers corresponding to Figure 3. Data from three independent experiments, mean and standard deviation (SD) are shown, Table S2: Raw values of virus titers corresponding to Figure 5. Data from three independent experiments, mean and standard deviation (SD) are shown. File S1: List of genes upregulated in KO cells.
