# Supplementary material for: Downregulation of A20 Expression Increases the Immune Response and Apoptosis and Reduces Virus Production in Cells Infected by the Human Respiratory Syncytial Virus
Source: Vaccines (Basel). 2020 Feb 24;8(1):100. doi: 10.3390/vaccines8010100 (PMC7157707; doi:10.3390/vaccines8010100)
Supplement: Supplementary file 1 [file vaccines-08-00100-s001.zip › Supplemental Fig. 1.pdf]

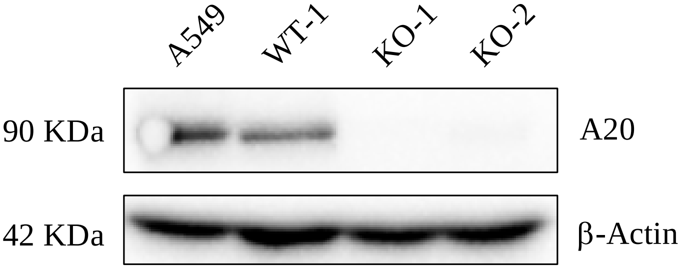

Supplemental Fig. 1. Two A20-knockout A549 cell lines (KO-1 and KO-2) were generated by using TALENs, and A20 expression was checked by Western blotting using specific antibodies. Uncloned wild-type cells (A549) an one wild-type cell clone (WT-1) were included as control.
