# Supplementary material for: Downregulation of A20 Expression Increases the Immune Response and Apoptosis and Reduces Virus Production in Cells Infected by the Human Respiratory Syncytial Virus
Source: Vaccines (Basel). 2020 Feb 24;8(1):100. doi: 10.3390/vaccines8010100 (PMC7157707; doi:10.3390/vaccines8010100)
Supplement: Supplementary file 1 [file vaccines-08-00100-s001.zip › Supplemental Fig. 2.pdf]

A549 (1)

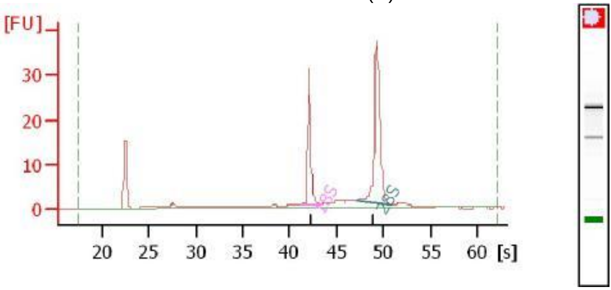

Overall Results for sample: **A549 (1)**

RNA Area:	120.3
RNA ConcenÜation:	1,078 ng/µl
rRNA Ratio [28s / 18s]:	2.0
RNA Integrity Number (RIN):	9.7 (B.02.08)
Result Flagging Color:	
Result Flagging Label:	RIN: 9.70

Fragment table for sample: **A549 (1)**

Name	Start Time (s)	End Time (s)	Area	% of total Area
18S	41.13	43.24	24.9	20.7
28S	47.12	50.85	50.9	42.3

KO-1 (1)

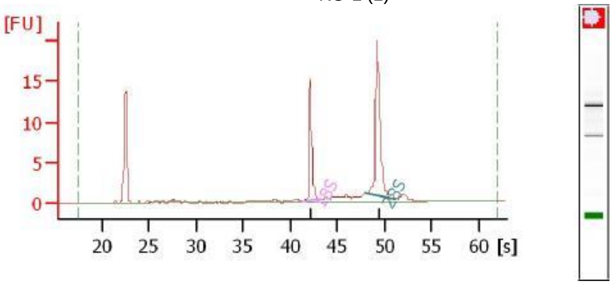

Overall Results for sample: **KO-1 (1)**

RNA Area:	55.8
RNA ConcenÜation:	500 ng/µl
rRNA Ratio [28s / 18s]:	1.9
RNA Integrity Number (RIN):	10 (B.02.08)
Result Flagging Color:	
Result Flagging Label:	RIN:10

Fragment table for sample: **KO-1 (1)**

Name	Start Time (s)	End Time (s)	Area	% of total Area
18S	41.27	43.29	11.1	19.9
28S	47.96	50.97	21.3	38.3

KO-2 (1)

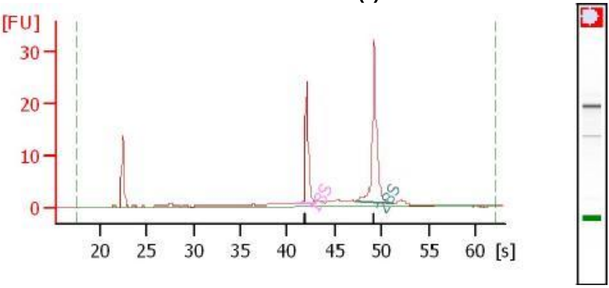

**Overall Results for sample:**	**KO-2 (1)**
**RNA Area:**	**87.2**
**RNA Concentration:**	**782 ng/µl**
**rRNA Ratio [28s / 18s]:**	**2.1**
**RNA Integrity Number (RIN):**	**9.6 (B.02.08)**
**Result Flagging Color:**	
**Result Flagging Label:**	**RIN: 9.60**
**Fragment table for sample:**	**KO-2 (1)**

Name	Start Time (s)	End Time (s)	Area	% of total Area
18S	40.86	42.89	17.4	20.0
28S	47.12	51.26	36.6	42.0

A549 (2)

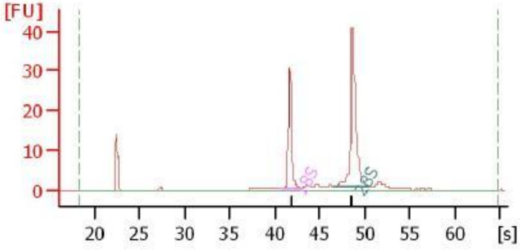

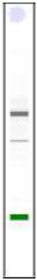

Overall Results for sample: A549 (2)

RNA Area:	111.4
RNA Concentration:	53 ng/µl
rRNA Ratio [28s / 18s]:	2.2
RNA Integrity Number (RIN):	10 (B.02.08)
Result Flagging Color:	
Result Flagging Label:	RIN:10

Fragment table for sample: A549 (2)

Name	Start Time (s)	End Time (s)	Area	% of total Area
18S	40.77	42.93	25.1	22.5
28S	46.60	50.54	53.9	48.4

KO-1 (2)

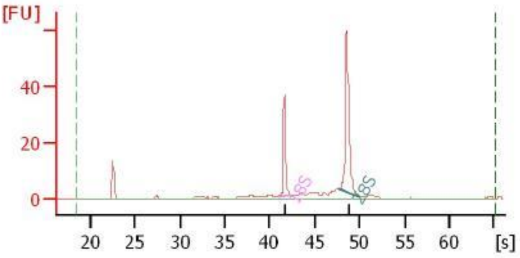

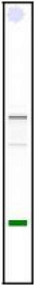

Overall Results for sample: KO-1 (2)

**RNA Area:**	**140.2**
**RNA ConcenÜation:**	**67 ng/µl**
**rRNA Ratio [28s / 18s]:**	**2.3**
**RNA Integrity Number (RIN):**	**9.6 (B.02.08)**
**Result Flagging Color:**	
**Result Flagging Label:**	**RIN: 9.60**

Fragment table for sample: KO-1 (2)

Name	Start Time (s]	End Time (s]	Area	% of total Area
18S	40.93	42.26	26.2	18.7
28S	47.69	50.06	59.9	42.7

KO-2 (2)

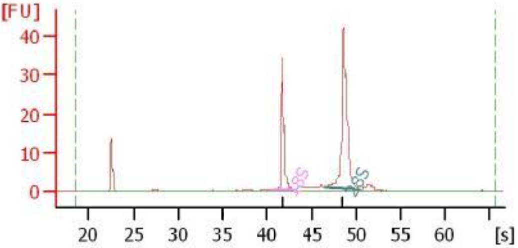

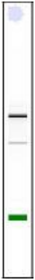

Overall Results for sample: KO-2 (2)

**RNA Area:**	**114.6**
**RNA Concentration:**	**54 ng/µl**
**rRNA Ratio [28s / 18s]:**	**2.0**
**RNA Integrity Number (RIN):**	**10 (B.02.08)**
**Result Flagging Color:**	
**Result Flagging Label:**	**RIN:10**

Fragment table for sample: KO-2 (2)

Name	Start Time (s)	End Time (s)	Area	% of total Area
18S	40.62	43.10	27.5	24.0
28S	46.52	50.42	55.5	48.4

A549 (3)

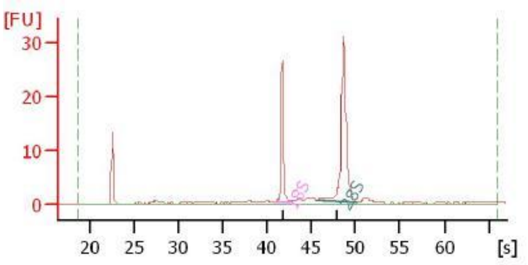

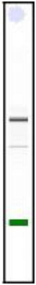

Overall Results for sample: **A549 (3)**

RNA Area:	86.9
RNA Concentration:	41 ng/µl
rRNA Ratio [28s / 18s]:	2.2
RNA Integrity Number (RIN):	10 (B.02.08)
Result Flagging Color:	
Result Flagging Label:	RIN:10

Fragment table for sample: **A549 (3)**

Name	Start Time (s)	End Time (s)	Area	% of total Area
18S	40.84	42.89	19.4	22.3
28S	45.72	50.17	41.7	48.0

KO-1 (3)

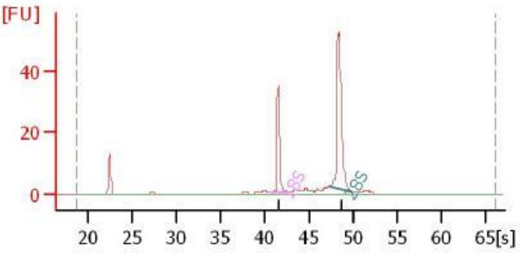

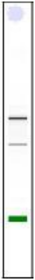

Overall Results for sample: KO-1 (3)

RNA Area:	118.1
RNA Concentration:	56 ng/µl
rRNA Ratio [28s / 18s]:	1.9
RNA Integrity Number (RIN):	10 (B.02.08)
Result Flagging Color:	
Result Flagging Label:	RIN:10

Fragment table for sample: KO-1 (3)

Name	Start Time (s)	End Time (s)	Area	% of total Area
18S	40.63	42.84	28.0	23.7
28S	47.40	50.13	54.4	46.0

KO-2 (3)

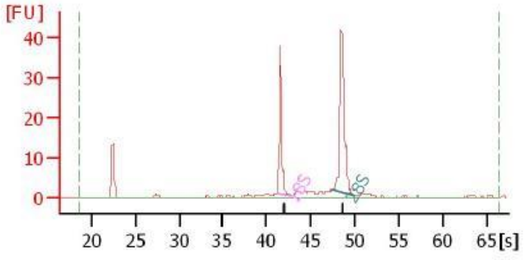

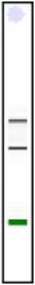

Overall Results for sample: **KO-2 (3)**

RNA Area:	111.4
RNA ConcenÜation:	53 ng/µl
rRNA Ratio [28s / 18s]:	2.0
RNA Integrity Number (RIN):	10 (B.02.08)
Result Flagging Color:	
Result Flagging Label:	RIN:10

Fragment table for sample: **KO-2 (3)**

Name	Start Time (s)	End Time (s)	Area	% of total Area

18S	41.25	42.79	26.4	23.7
28S	47.36	49.86	52.9	47.5
