# Supplementary material for: Downregulation of A20 Expression Increases the Immune Response and Apoptosis and Reduces Virus Production in Cells Infected by the Human Respiratory Syncytial Virus
Source: Vaccines (Basel). 2020 Feb 24;8(1):100. doi: 10.3390/vaccines8010100 (PMC7157707; doi:10.3390/vaccines8010100)
Supplement: Supplementary file 1 [file vaccines-08-00100-s001.zip › Supplemental Fig. 3.pdf]

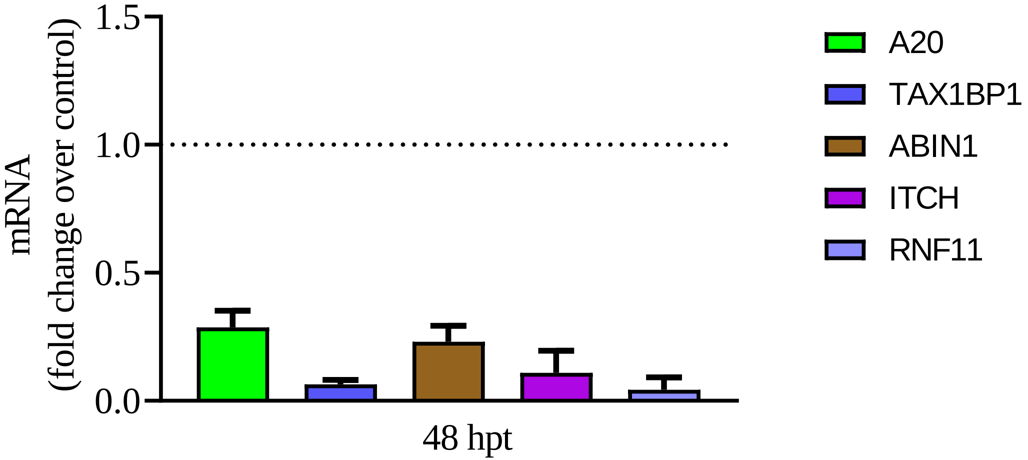

Supplemental Fig. 3. Gene silencing of A20, TAX1BP1, ABIN1, ITCH and RNF11 genes. Cells were transfected with the corresponding siRNAs (A20, TAX1BP1, ABIN1, ITCH or RNF11), infected with HRSV 24 h later and cells were harvested at 24 hpi (48 h post-transfection). Levels of the different mRNAs (A20, TAX1BP1, ABIN1, ITCH or RNF11) were quantified by qRT-PCR and represented as fold-over mRNAs expressed in cells transfected with a control siRNA. Data are represented as the mean and standard deviation from three independent experiments.
