# Supplementary material for: Downregulation of A20 Expression Increases the Immune Response and Apoptosis and Reduces Virus Production in Cells Infected by the Human Respiratory Syncytial Virus
Source: Vaccines (Basel). 2020 Feb 24;8(1):100. doi: 10.3390/vaccines8010100 (PMC7157707; doi:10.3390/vaccines8010100)
Supplement: Supplementary file 1 [file vaccines-08-00100-s001.zip › Supplemental Fig. 4.pdf]

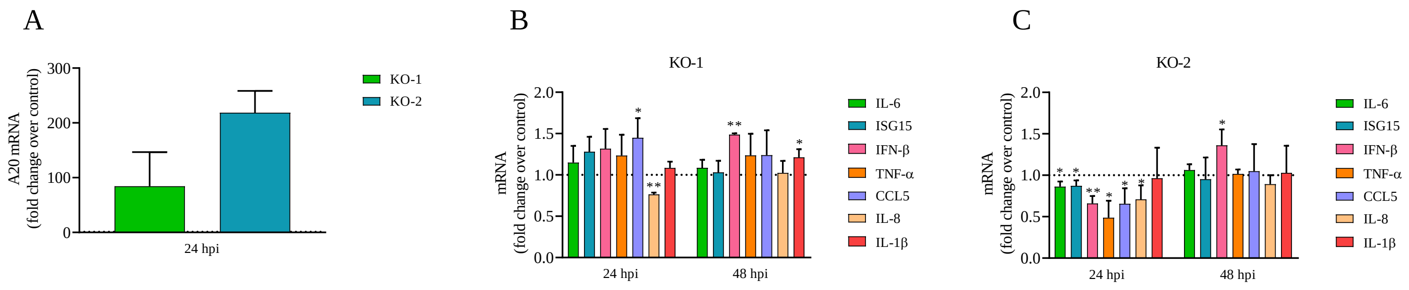

Supplemental Fig. 4. Overexpression of A20 in KO lines. KO-1 and KO-2 cells were transfected with a plasmid encoding A20, infected with HRSV 24 h later, and cells were harvested at 24 and 48 hpi. Levels of the different mRNAs were quantified by qRT-PCR and represented as fold-over mRNAs expressed in cells transfected with a control plasmid (empty vector). (A) mRNA levels of A20. (B) mRNA levels of IL-6, ISG15, IFN-β, TNF-α, CCL5, IL-8 and IL-1β in KO-1 cells. (C) mRNA levels of IL-6, ISG15, IFN-β, TNF-α, CCL5, IL-8 and IL-1β in KO-2 cells. Data are represented as the mean and standard deviation from three independent experiments. Comparisons between groups were done by using the t-test: *, $p < 0.05$ and **, $p < 0.01$.
