# Supplementary material for: Downregulation of A20 Expression Increases the Immune Response and Apoptosis and Reduces Virus Production in Cells Infected by the Human Respiratory Syncytial Virus
Source: Vaccines (Basel). 2020 Feb 24;8(1):100. doi: 10.3390/vaccines8010100 (PMC7157707; doi:10.3390/vaccines8010100)
Supplement: Supplementary file 1 [file vaccines-08-00100-s001.zip › Supplemental Fig. 5.pdf]

A

	Mean (SD)
A20	423.1 (276.7)
TAX1BP1	58.28 (28.69)

B

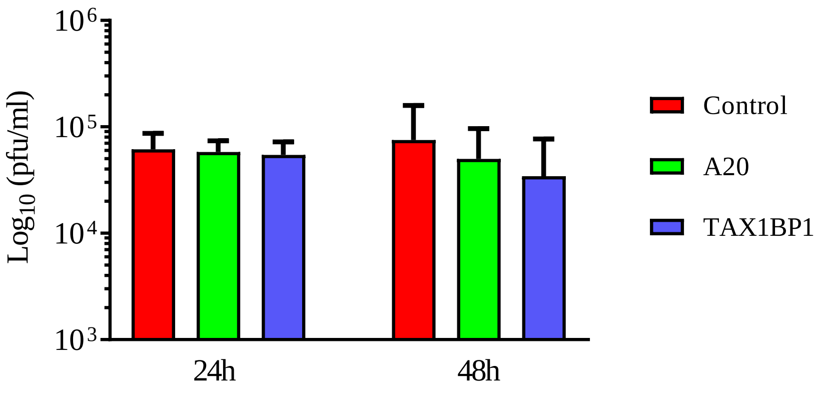

C

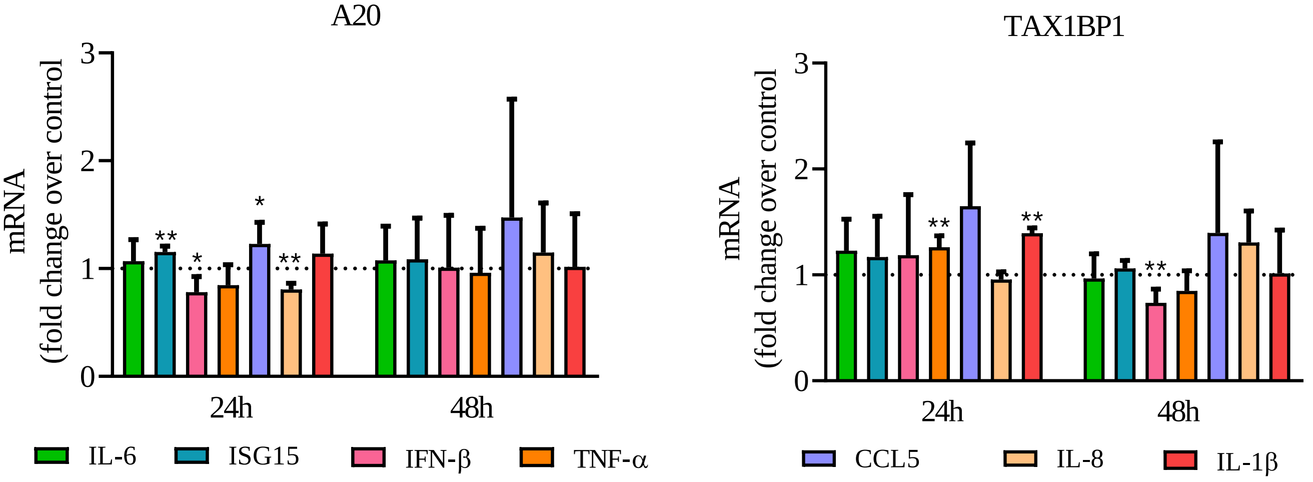

D

	**Mean (SD)**
A20	250.90 (50.95)
TAX1BP1	16.73 (2.037)
ABIN1	318.50 (41.70)

E

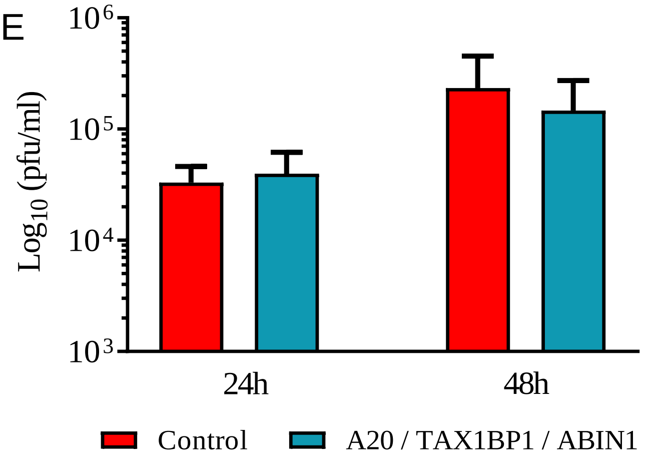

F

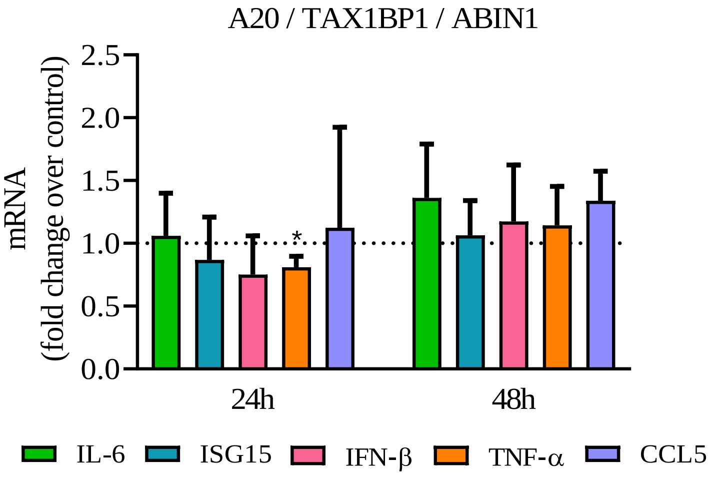

Supplemental Fig. 5. HRSV titers and immune gene expression in cells transfected with individual plasmids encoding A20 or TAX1BP1 (A-C) or with a mixture of plasmid encoding A20, TAX1BP1 and ABIN1 (D-F). Cells were transfected with the corresponding plasmids, infected 24h later with HRSV and harvested at 24 hpi (48 h post-transfection). Levels of the different mRNAs (A, C, D, F) were quantified by qRT-PCR and represented as fold-over mRNAs expressed in cells transfected with a control plasmid. Data are represented as the mean and standard deviation from three independent experiments. Comparisons between groups were done by using the t-test: *, $p < 0.05$ and **, $p < 0.01$.
