# Supplementary material for: Downregulation of A20 Expression Increases the Immune Response and Apoptosis and Reduces Virus Production in Cells Infected by the Human Respiratory Syncytial Virus
Source: Vaccines (Basel). 2020 Feb 24;8(1):100. doi: 10.3390/vaccines8010100 (PMC7157707; doi:10.3390/vaccines8010100)
Supplement: Supplementary file 1 [file vaccines-08-00100-s001.zip › Supplemental Tables.docx]

**Supplemental Table S1.** Raw values of virus titers corresponding to Figure 3. Data from three independent experiments, mean and standard deviation (SD) are shown.

|  | Control 24h | A20 24h | TAX1BP1 24h | ABIN1 24h | ITCH 24h | RNF11  24h | Control 48h | A20 48h | TAX1BP1 48h | ABIN1  48h | ITCH 48h | RNF11  48h |
| --- | --- | --- | --- | --- | --- | --- | --- | --- | --- | --- | --- | --- |
| 1 | 4.8 E4 | 5.9 E4 | 6.1 E4 | 4.5 E4 | 5.3 E4 | 3.3 E4 | 2.8 E6 | 9.3 E5 | 5.2 E5 | 1.3 E6 | 3.7 E5 | 1.8 E6 |
| 2 | 8.9 E4 | 4.9 E4 | 8.1 E4 | 4.5 E4 | 3.8 E4 | 3.4 E4 | 1.9 E6 | 2.7 E5 | 2.9 E5 | 1.0 E6 | 3.2 E5 | 1.0 E6 |
| 3 | 1.7 E4 | 7.8 E4 | 8.8 E4 | 4.6 E4 | 5.0 E4 | 3.5 E4 | 2.5 E6 | 1.4 E6 | 4.6 E5 | 1.0 E6 | 3.1 E5 | 1.5 E6 |
| Mean | 5.1 E4 | 6.2 E4 | 7.7 E4 | 4.5 E4 | 4.9 E4 | 3.4 E4 | 2.4 E6 | 8.8 E5 | 4.3 E5 | 1.1 E6 | 3.3 E5 | 1.4 E6 |
| SD | 3.6 E4 | 1.5 E4 | 1.4 E4 | 5.0 E2 | 1.1 E4 | 1.1 E3 | 4.4 E5 | 5.8 E5 | 1.2 E5 | 1.6 E5 | 3.3 E4 | 3.9 E5 |

**Supplemental Table S2.** Raw values of virus titers corresponding to Figure 5. Data from three independent experiments, mean and standard deviation (SD) are shown.

|  | A549 24h | WT-1 24h | KO-1 24h | KO-2 24h | A549 48h | WT-1 48h | KO-1 48h | KO-2 48h |
| --- | --- | --- | --- | --- | --- | --- | --- | --- |
| 1 | 7.5 E4 | 9.8 E4 | 1.2 E5 | 4.7 E4 | 2.5 E6 | 3.2 E6 | 8.8 E5 | 8.8 E5 |
| 2 | 6.3 E4 | 5.8 E4 | 7.2 E4 | 3.5 E4 | 1.3 E6 | 2.5 E6 | 7.8 E5 | 3.8 E5 |
| 3 | 6.0 E4 | 6.3 E4 | 1.1 E5 | 5.0 E4 | 1.1 E6 | 2.2 E6 | 8.8 E5 | 9.7 E5 |
| Mean | 6.6 E4 | 7.3 E4 | 1.0 E5 | 4.4 E4 | 1.6 E6 | 2.6 E6 | 8.5 E5 | 7.4 E5 |
| SD | 7.9 E3 | 2.2 E4 | 2.5 E4 | 7.9 E3 | 7.5 E5 | 5.1 E5 | 5.8 E4 | 3.2 E5 |
